# Antibodies against CD20 or B-Cell Receptor Induce Similar Transcription Patterns in Human Lymphoma Cell Lines

**DOI:** 10.1371/journal.pone.0016596

**Published:** 2011-02-18

**Authors:** Andreas Franke, Gerhard J. Niederfellner, Christian Klein, Helmut Burtscher

**Affiliations:** 1 Pharma Research and Early Development, Roche Diagnostics GmbH, Penzberg, Germany; 2 Pharma Research and Early Development, Roche Glycart AG, Schlieren-Zürich, Switzerland; University of Barcelona, Spain

## Abstract

**Background:**

CD20 is a cell surface protein exclusively expressed on B cells. It is a clinically validated target for Non-Hodgkin's lymphomas (NHL) and autoimmune diseases. The B cell receptor (BCR) plays an important role for development and proliferation of pre-B and B cells. Physical interaction of CD20 with BCR and components of the BCR signaling cascade has been reported but the consequences are not fully understood.

**Methodology:**

In this study we employed antibodies against CD20 and against the BCR to trigger the respective signaling. These antibodies induced very similar expression patterns of up- and down-regulated genes in NHL cell lines indicating that CD20 may play a role in BCR signaling and vice versa. Two of the genes that were rapidly and transiently induced by both stimuli are CCL3 and CCL4. 4 hours after stimulation the concentration of these chemokines in culture medium reaches a maximum. Spleen tyrosine kinase Syk is a cytoplasmic tyrosine kinase and a key component of BCR signaling. Both siRNA mediated silencing of Syk and inhibition by selective small molecule inhibitors impaired CCL3/CCL4 protein induction after treatment with either anti-CD20 or anti-BCR antibodies.

**Conclusion:**

Our results suggest that treatment with anti-CD20 antibodies triggers at least partially a BCR activation-like response in NHL cell lines.

## Introduction

Activation of B cells is a tightly controlled process. One major component of these complex control mechanisms is the B cell antigen receptor (BCR) [1], a multimeric complex of membrane proteins with at least two immunoglobulin molecules together with CD79α/β in the core-unit and many accessory proteins [2]. The complexity of the downstream signaling events can lead to distinct outcomes (development, differentiation, apoptosis or activation of B lymphocytes), depending on the maturation state of the cell, magnitude and duration of activation, and modulating signals from other pathways (eg. CD40, CD19, CD45, CD22, PIR-B, CD32/FcγIIB) [3]. B cells that escape from this control can give rise to leukemia or lymphoma [4]. In recent years the anti-CD20 antibody rituximab has led to major improvements in the treatment of NHL and rheumatoid arthritis [5]. Besides riuximab which is a so called type I anti-CD20 antibody, type II antibodies are scrutinized at the moment. In addition to ADCC and CDC, mediated via the Fc-part of an anti-CD20 antibody, mostly the so called type II anti-CD20 antibodies also cause direct cell death by binding CD20 [6] - but the exact contribution of these different molecular mechanisms to efficacy is not yet fully understood [7], [8].

CD20 (official gene symbol is MS4A1) is a B cell specific, tetraspanning membrane protein of unknown function without a known ligand. Several observations point to an interrelation with the BCR: In the absence of rescuing/anti-apoptotic signals B cells in culture undergo apoptosis/cell death after crosslinking BCR as well as after crosslinking CD20 [9]–[14]. Immunofluorescence experiments showed that BCR and CD20 co-localize in lipid rafts upon treatment with type I CD20 antibodies [15]. There also seems to be a common connection with calcium flux [16], [17]. Similar phospho-protein patterns have been described, which led to the speculation that CD20 may “hijack” BCR signaling components [16]. Moreover, direct physical coupling of CD20 and BCR has been reported [18].

Although there are a few other examples of agonistic antibodies triggering signal cascades is not a common feature of antibodies. Therefore it is noteworthy that anti-CD20 and anti-BCR antibodies may activate interfering signal transduction [19], [20]. A signaling cascade at least in part common to BCR and CD20 has also strongly been implicated by the facts that a survival factor for B cells called BAFF (TNFSF13B) is able to block apoptosis mediated by both [21] and that expression of six genes changed similarily after treatment with anti-CD20 and BCR antibodies [22].

The goal of this study was to test on the whole transcriptome level whether concordant gene expression changes occur after BCR activation and anti-CD20 antibody treatment of human lymphoma cells.

## Results

### Effect of anti-BCR treatment on the level of transcription

Because expression of IgM (immunoglobulin M) is a hallmark of B cells and most lymphoma cell lines contain IgM as immunoglobulin part of the BCR [21], [23] anti-IgM antibodies are generally used for activation of the BCR *in-vitro* [3], [24]–[27]. There are some cell lines (eg. SUDHL4 [16], DOHH2 [19]), however, that are reported to utilize IgG (immunoglobulin G) instead of IgM. The cell lines used in this study (Z138, OciLy18, REC1 and SUDHL4) were all treated with both anti-IgM- and anti-IgG antibodies to trigger B cell receptor. To trigger CD20 signaling we applied anti-CD20 antibodies called rituximab and LT20, respectively.

As Fcγ receptors can interfere with the BCR signaling pathway [28], we included LT20 containing a murine Fc-part and F(ab')_2_-fragments of anti-IgM antibodies to check, if there was an influence of the human Fc-part of the applied whole antibodies capable of binding to Fcγ receptors.

Of the four cell lines tested REC1 responded most strongly to anti-IgM and anti-IgG antibody treatment in terms of numbers of deregulated genes, while OciLy18 and Z138 showed fewer gene expression changes. SUDHL4 responded strongly to anti-IgG antibody whereas after treatment with anti-IgM antibodies almost no significant changes in gene expression occurred (Table 1). FACS (fluorescence activated cell sorting) analysis confirmed that these cells only express IgG but not IgM on the cell surface. This is consistent with previous reports [16].

**Table 1:** Number of up and down regulated genes.

Treatment/cells	Rituximab	LT20	Anti-IgM-F (ab')2	Anti-IgM	Anti-IgG	Isotype/human IgG
OciLy18	1	6	434	383	141	0
Z138	10	15	437	283	506	1
REC1	108245 (F (ab)2)	445	1412	1190	1028	70
SUDHL4	509	637	40	4	680	0

Filter criteria: mean >100, call >0.5, |fold change| >2.

These experiments showed that both anti-IgM and anti-IgG treatments were able to trigger specific transcription changes.

### Comparison of transcriptional changes after treatment with anti-IgM and anti-IgG antibodies

As expected treatment of Ocily18 with anti-IgM antibodies or anti-IgM F(ab')_2_-fragment showed extensive overlap with regard to gene expression changes but also with treatment with anti-IgG antibodies (Table 2).

**Table 2:** Overlapping genes between different treatments.

Treatment/cells	Rituximab vs. LT20	Anti-IgM vs. Anti-IgM-F (ab')2	Anti-IgM/F (ab)2 vs. anti-IgG	Anti-BCR vs. anti-CD20
OciLy18	NA	312	102	NA
Z138	NA	246	181	NA
REC1	98*	977	768	89
SUDHL4	436	NA	NA	342**

*Intersection of genes deregulated by rituximab, rituximab-F(ab')_2_ and LT20.

**Only anti-IgG antibody as anti-BCR treatment.

Similar results were obtained with the cell line Z138. While Z138 cells in terms of number of gene expression changes responded equally well to cross-linking of IgG and IgM, the OciLy18 cells were three-fold more responsive to anti-IgM versus anti-IgG treatment. As a consequence, the overlap of the transcriptional changes induced by the two treatments was larger in the latter cells. In REC1 cells due to even stronger gene expression responses the overlap was even more extensive.

The large overlap in the gene expression responses to the whole anti-IgM and the anti-IgM F(ab')_2_-fragment observed for all three cell lines attests to the high technical and biological reproducibility of the anti-IgM response. In general fewer genes responded to anti-IgM compared to anti-IgM-F(ab')_2_ treatment in these three cell lines. This might reflect inhibitory effects exerted by the Fc part via binding to FCGR2.

The higher variability of the anti-IgG response of the three cell lines correlated with the differences in the levels of cell surface exposed IgG molecules. Although all antigen-binding membrane immunoglobulins, irrespective of the isotype, associate with the CD79α/β chains, it has been previously shown that BCRs containing IgM cytoplasmic tails are regulated by co-receptor CD22, whereas those containing IgG cytoplamic tails are not [29], [30]. In agreement with this, in all cell lines that represent an immature B-cell state and still express IgM type BCRs the two transcriptional responses only partly overlapped. SUDHL4 cells represent a memory B-cell state and only respond to anti-IgG but not to anti-IgM treatment with gene expression changes.

Expression of CD22 and other potential co-receptors of the BCR as well as markers of differentiation were determined by flow-cytometry (and confirmed by mRNA expression) to stage the cell lines studied (Table 3 and 4). CD22 is expressed in all cell lines except Z138. The expression pattern of other co-receptors like CD10, CD27, CD138 also points to a plasmablastoid phenotype of Z138. CD27 expression indicates ongoing differentiation but is missing in Z138, whereas SUDHL4 expresses CD27 confirming the memory B-cell state of these cells.

**Table 3:** Antigen expression determined by FACS analysis.

CDs/cells	OciLy18	Z138	REC1	SUDHL4
CD19	++++	+	+++	+++++
CD20	+	++	+++	++++
CD21	+	+	++	+
CD22	+	-	+	+
CD27	++	+	+	+++++
CD34	+	+	+++	++
CD38	+++	++++	++	+++++
CD138	++	++++	+	+
IgD	-	+	++	-
IgM	++	++	+++	-
CD79A	+	++	++	+
CD79B	+	+	+	+

**Table 4:** Antigen expression determined by Affymetrix analysis: mean expression values of untreated cells.

Gene name	OciLy18	Z138	REC1	SUDHL4	Description
CD10	268	*	79	973	membrane metallo-endopeptidase
CD19	752	273	972	333	cd19 molecule
CD20	3472	3458	3988	4324	membrane-spanning 4-domains, subfamily a, member 1
CD21	*	*	814	*	CD21 complement component (3d/epstein barr virus) receptor 2
CD22	758	*	311	1200	cd22 molecule
CD24	1709	559	2007	123	cd24 molecule
CD27	559	*	*	1603	cd27 molecule
CD34	*	*	*	*	cd34 molecule
CD38	164	521	156	397	cd38 molecule
CD138	*	119	*	*	syndecan 1
IgD	188	286	*	*	immunoglobulin heavy constant delta
IgM	4933	4005	3822	*	immunoglobulin heavy constant mu
CD79A	1046	745	2653	1114	cd79a molecule, immunoglobulin-associated alpha
CD79B	2322	425	1289	653	cd79b molecule, immunoglobulin-associated beta

*below background.

In these cell lines anti-IgM antibodies triggered a BCR signaling like cascade leading to characteristic gene regulation responses. A very similar/overlapping transcription pattern was triggered by anti-IgG antibodies. This allowed using anti-IgG antibodies as an alternative trigger for BCR activation in a cell line that lacks IgM but exposes IgG, as is the case with SUDHL4.

### Transcription changes induced by anti-CD20 antibodies

Although Z138 and OciLy18 express CD20 on their cell surface (Table 3) these cell lines did not or only very little respond to anti-CD20 antibodies in terms of transcriptional changes. This indicates that CD20 expression alone at the cell surface is not sufficient to trigger substantial transcriptional responses.

In SUDHL4 and REC1 anti-CD20 treatment elicited extensive changes in transcription: Treating SUDHL4 with rituximab and LT20 resulted in an extensive overlap of deregulated genes with only very few unique deregulated genes (Table 2). The remarkable concordance between the transcription patterns of the two different type I anti-CD20 antibodies strongly indicates that the observed effects were target-mediated. In REC1 cells transcriptional changes after treatment with rituximab or its F(ab')_2_-fragment were less pronounced than changes after LT20 treatment. Not unexpectedly, the same genes responded to treatment with rituximab and its F(ab')_2_-fragment. These genes are a subset of the larger panel that was deregulated by LT20 treatment.

These results show that in 2 out of 4 cell lines studied specific transcription changes were induced by anti-CD20 treatment, independent of the isotype of antibody used.

### Comparison of expression changes induced by anti-CD20 and anti-BCR treatment

Having checked the consistency of the treatments with antibodies against either CD20 or BCR we compared anti-CD20 with anti-BCR treatment.

**Figure 1:**
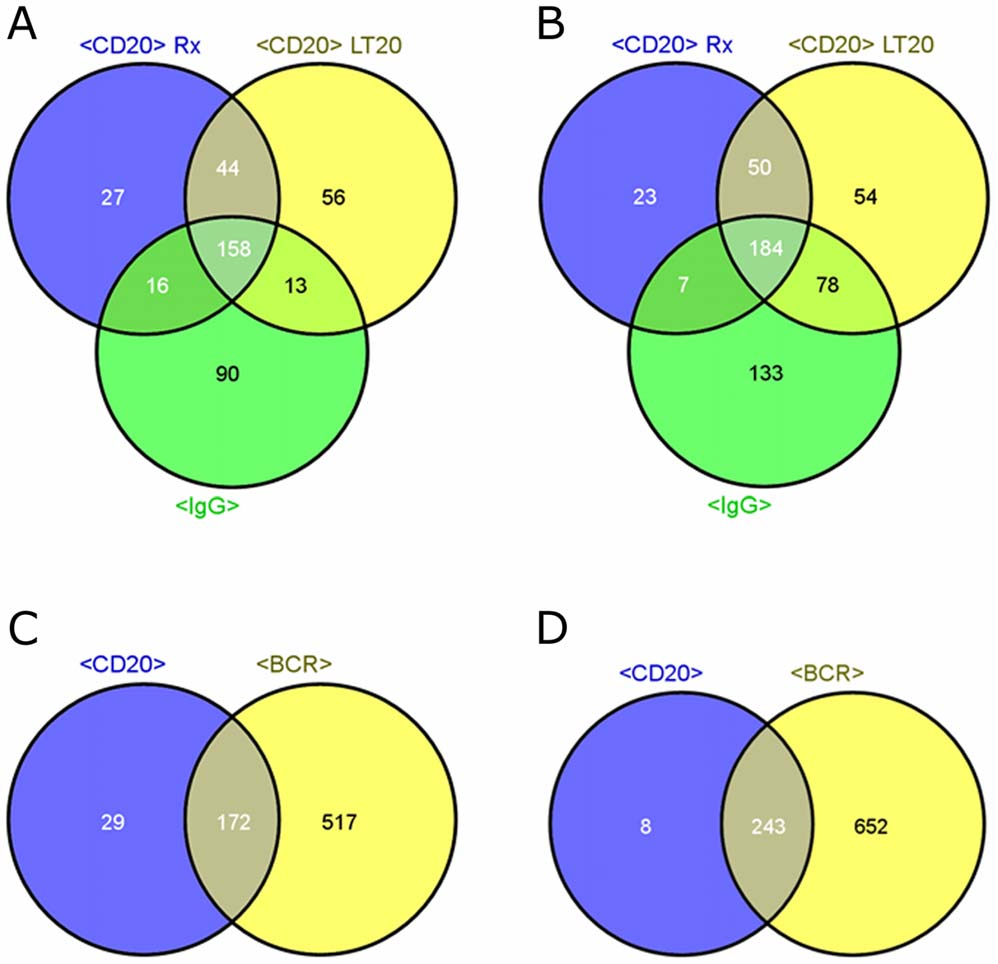
Comparison of genes deregulated by different antibody treatments. Venn diagrams for numbers of up- (A) or down- (B) regulated genes in SUDHL4 after a 4 h incubation with 10 µg/ml anti-BCR antibody (anti-IgG) and anti-CD20 antibodies (Rituximab, LT20) and Venn diagrams for up- (C) or down- (D) regulated genes in REC1 after treatment with anti-BCR antibodies (anti-IgG and anti-IgM, respectively) and anti-CD20 antibodies (Rituximab, Rx-F(ab')_2_, LT20).

With SUDHL4 the transcription patterns overlapped in 342 deregulated genes (Figure 1A and B; Table 2, Individual genes are listed in Table S1.) i.e. 48% of the anti-CD20 pattern were covered by the BCR pattern. The corresponding comparison for REC1 results in an overlap of 92% (Figure 1C and D, 415 genes overlap with anti-BCR treatment out of 452 deregulated genes after anti-CD20 treatment. Individual genes for intersection of union lists are listed in Table S2).

**Figure 2:**
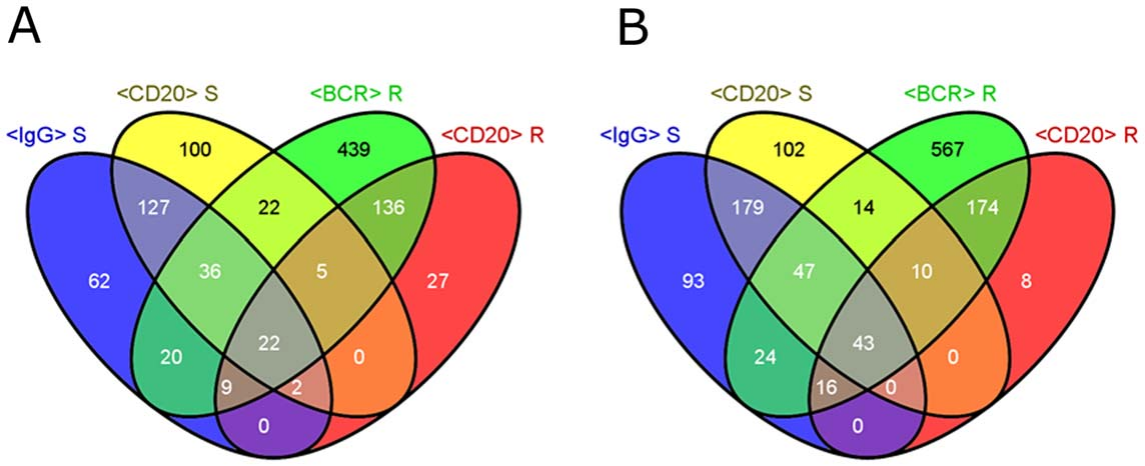
Comparison of anti-BCR and anti-CD20. Venn diagrams of “Union lists” of up- (A) or down- (B) regulated genes after different treatments. Cells were incubated with 10 µg/ml antibody for 4 h. Lists of similar treatments were united to “Union lists” for CD20 or BCR treatment for each cell line R = REC1, S = SUDHL4.

Besides comparing anti-CD20 and anti-BCR treatments in REC1 and SUDHL4 separately we also analyzed which changes were common to both cell lines. The overlaps of the union lists are rather small: 65 genes were deregulated in common compared to cell line intersections of 306 and 310 genes exclusively deregulated in SUDHL4 and REC1, respectively (Figure 2, Individual genes are listed in Table S3).

This analysis suggested that the transcription changes induced by anti-BCR and anti-CD20 antibodies partially overlap but that the (whole) response pattern is highly cell line specific.

### Comparison of activation patterns of different cell lines

**Figure 3:**
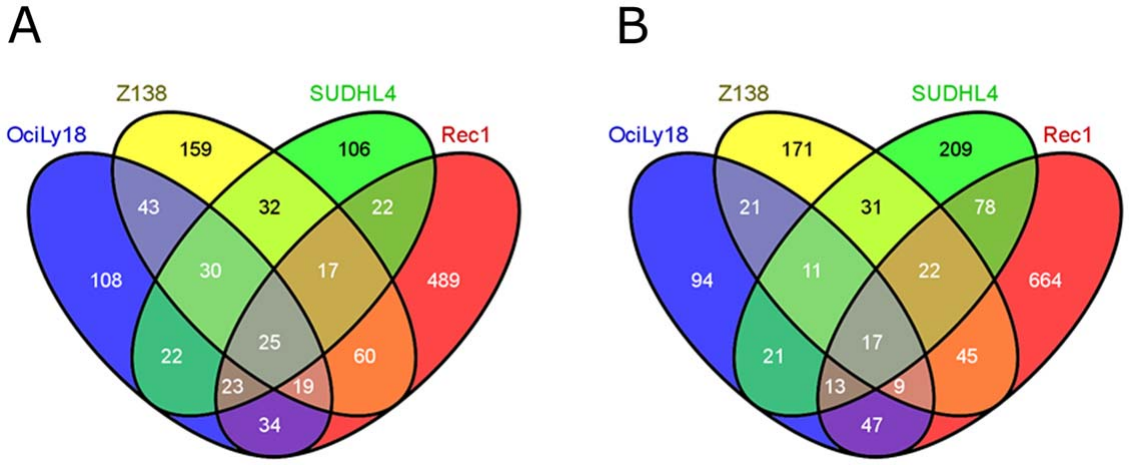
Comparison of anti-BCR treatments in all cell lines studied. Venn diagrams of “Union lists” ( = Union of lists for similar treatments) for anti-BCR treatment of up- (A) or down- (B) regulated genes in OciLy18, Z138, SUDHL4 and REC1.

Given the extensive overlaps between the responses to anti-BCR and anti-CD20 treatments within each of the two cell lines, and yet the small overlaps between both cell lines, we compared the BCR transcription patterns across all four cell lines (Figure 3). Here again the overlaps were rather small and the proportion of genes uniquely regulated in a given cell line was much higher. Only 25 up- and 17 down-regulated genes were common in all four cell lines (Individual genes are listed in Table S4). The deregulated genes contained in this overlap were analyzed further with respect to their functional context (Table 5).

**Table 5:** Functional clustering of genes deregulated by anti-BCR antibodies in cell lines OciLy18, Z138, REC1 und SUDHL4.

Pathway, Functional Cluster	Genes
BCR signaling/B cell activation, Immune response	DAPP1, ETS1, GEM, KLF6, PIK3R3, RFXAP, VPREB3,
Transcription	ETS1, IER2, MAFF, SSBP3, ZFP36L, ZFP362
Apoptosis, cellular response to stress, cellular response to unfolded protein	RNF144B, SGK1, SQSTM1, SRGN, PPP1R15A
MAPK signaling/p38 Kinase signaling	DDIT3, MAPK3, SGK1, TESK1
N-Glycan biosynthesis, Other glycan degradation/Spingolipid metabolism/Lysosome	MGAT4B, NEU1 ( = Sialidase1)
Mitotic cell cycle, JAK/STAT signaling/Acute myeloid leukaemia	SAC3DC1, OIP5, PIM1
Cytoskeleton/cell-substrate –junction	APBB1IP, MICAL1
Fc gamma R mediated phagocytosis	VASP
NFkB signaling	NFKBID
Antigen Processing and presentation	RFXAP
Cellular homeostasis	GLRX
GPCR signalling	RGS1
Glucose uptake	SLC2A3
Base excision repair	NEIL3
mRNA processing, Dicer pathway, mRNA cleavage	EIF2C2
TGF beta signalling	C5orf13

To identify the functional clusters behind the genes influenced by anti-CD20 treatment we conducted the analogous comparison and analysis for GO annotation, Panther, Biocarta and KEGG pathways (Table 6) as described above for the BCR response genes. We found that many deregulated genes are associated with cell death, stress signaling, BCR activation, immune response, and development of hematopoietic cells.

**Table 6:** Functional clustering of genes deregulated by anti-CD20 antibodies in SUDHL4 cells und REC1 cells (KEGG-, Panther- und Biocarta, GO terms).

Pathway/Functional Cluster	Genes
Apoptosis, Cell death	ADRB2, BCL2L11, BCL2A1, CDKN2C, ETS1, RNF144B, SRGN, TXNIP, TNFAIP3,
BCR, immune response	CD79B, ETS1, FCGR2B, DAPP1, BLK, VPREB3
MAPK signaling, p38 signaling	DUSP5, RPS6KA5, C21orf7, PTPN18, TRIB1, TXNIP
Transcription regulation, regulation of RNA stability	ETS1, IRF2BP2, SSBP3, (TRIM22), ZFP36L1
Calcium signaling, response to calcium ion	ATP2A3, KCNMB4
Fcgamma mediated phagocytosis	FCGR2B, NCF1, VASP
Cell cycle, cell division	CDKN2C, TXNIP
ECM receptor interaction, cell adhesion	HMMR, ITGB7
Endocytosis	RAB11FIP4
Cellular response to stress	INSIG1, TXNIP, TRIB1
NFkB pathway	NLRC3, NDFIP2, TNFAIP3
Arachidonic acid metabolism	ALOX5, PTGER4
GPCR signaling	RABGAP1L
P53 signaling	DDB2, CDKN2C
Proteolysis	NAPSB, SERPINA9
Cytoskeleton, intracellular trafficking	APBB1IP, SNX8, DAAM1
RNA degradation	EXOSC4
Pathways in Cancer	ETS1
Pyrimidine metabolism	UPP1
Aldosteron regulated sodium absorption	SGK1
NOD-like receptor signaling	TNFAIP3
T cell activation	TRVD2, TRA
TGF beta signaling	C21orf7

**Figure 4:**
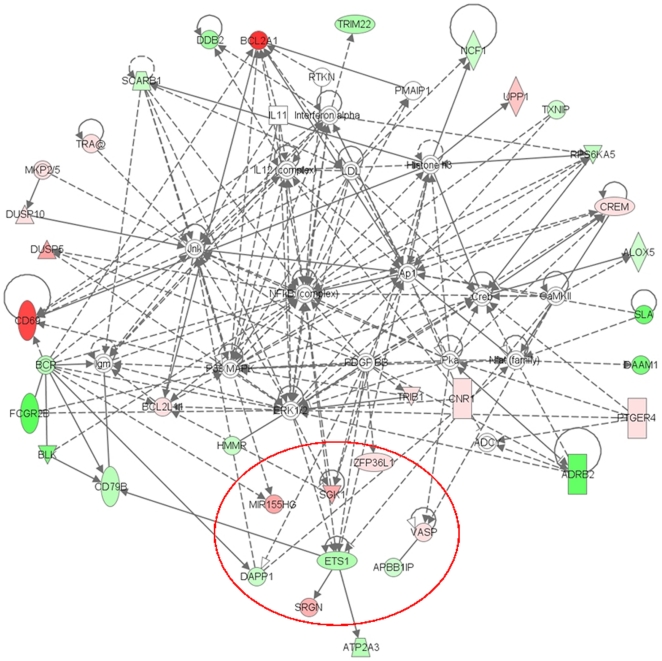
Interactions between deregulated genes. IPA generated network of interactions between genes deregulated by anti-CD20 antibodies in SUDHL4 and REC1. The red circle marks genes which are also deregulated by all anti-BCR treatments. Interestingly this unbiased analysis displays deregulations of BCR components and the downstream signaling pathway.

Despite the differing transcription patterns of the two cell lines responding to anti-CD20 and of all four cell lines responding to anti-BCR a few genes were affected by all treatments. Of these 5 were up- and 8 were down-regulated. They comprise transcription factors, adaptor proteins for signaling cascades, a ubiquitin ligase and a stress kinase – proteins that are involved in the NFκB (Nuclear factor kappa B) signaling pathway, cellular stress and apoptosis (Table 7 and Figure 4). A noteworthy aspect is the down-regulation of genes associated with the BCR signaling pathway: DAPP1, ETS1 and VPREB3. Another interesting finding was the induction of BIC - a gene locus for miRNA155.

**Table 7:** Genes that are common deregulated in all four cell lines by anti-BCR antibodies and in SUDHL4 and REC1 by anti-CD20 antibodies.

Gene Symbol	Function/Description
Up regulated genes	
BIC	- Genlocus for miRNA-155- is regulated by PKC and NFKB after BCR stimulation [36]
SGK1	- a Serine/Threonine kinase, important for cellular stress response- regulated GSK3 and binds IKK (NFkB Pathway)
SRGN	- is associated with Granzyme/Perforine complex, perhaps a mediator of apoptosisi- regulates secretion of TNFα
VASP	- a adaptor protein, involved in intracellular signal transduction, that regulates interaction between integrines and intercellular matrix- is regulated by PKA and PKG
ZFP36L1	- a Zinc finger transcription factor, activated by cellular stess- is a early response gene
Down regulated genes	
APBB1IP	- plays role in signaltransduction of Ras to remodel the actin cyto skeleton- interacts with VASP- plays role in TCR activation
C5orf13	- open reading frame without known funtion
DAPP1	- adaptor protein, regulates JNK, RAC1 and MAPK signaling cascades
ETS1	- a transcription factor, plays role in stem cell development, cellular senescence and death- associated with BCR stimulation [23]- regulates CDKN1A (p21, cell cycle inhibitor), MDM2 (p53 antagonist) and MMPs
GLRX	- a thioltransferase, important in NFkB signaling pathway
RNF144B	- a ring finger protein; E3 ubiquitin ligase, avoiding spontaneous apoptosis [47] (BRDC2 = RNF144B)- is regulated by p53- ubiquitinates CDKN1A
SSBP3	- a single strand binding protein, transcription regulator
VPREB3	- associated with µ chain in pre-B cell receptor synthesis, supposed to be involved in its transport

The gene expression results suggested that antibodies against CD20 and antibodies against BCR share common signaling pathway components.

### Influence of SYK inhibition or siRNA mediated SYK down-regulation on both anti-CD20 and anti-BCR treatment

Based on the findings outlined above, we hypothesized that the CD20 signaling cascade also contains the kinase SYK. In the BCR pathway, SYK acts downstream of the signaling molecules CD79α/β and upstream of NFκB or other signaling cascades towards the nucleus.

**Figure 5:**
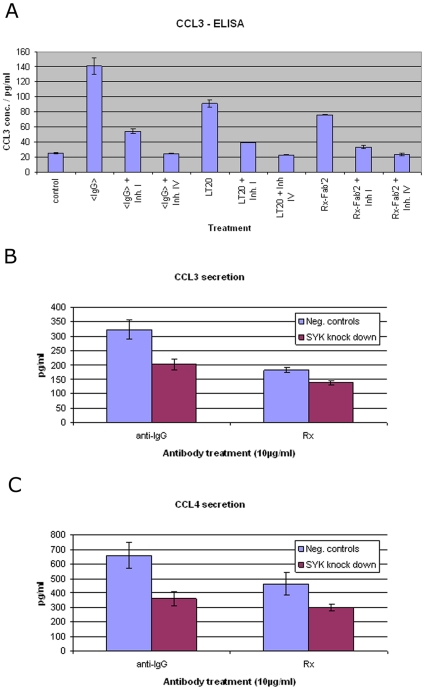
Repression of chemokine CCL3/4 induction caused by Syk inhibition and silencing. (A) CCL3 secretion in SUDHL4 induced by anti-IgG antibodies and anti-CD20 antibodies and its inhibition by Syk inhibitors I and IV (Calbiochem) at 1 µM and 0,32 µM respectively. Bars represent mean of three replicates, including standard deviation (<IgG>  =  anti-IgG antibody, Inh. I  =  Syk inhibitor I, Inh. IV =  Syk inhibitor IV, LT20  =  murine anti-CD20 antibody, Rx-Fab'2  =  F(ab')_2_ fragment of rituximab). Inhibition of CCL3 (B) and CCL4 (C) secretion in SUDHL4 induced by anti-IgG antibodies or rituximab after siRNA-mediated Syk silencing. Results represent at least three independent experiments. Students t-test for “Negative controls” vs. “SYK-knockdown”: for CCL3 secretion after anti-IgG treatment p = 6.8*10^−7^, Rx treatment p = 7.4*10^−3^. For CCL4 secretion after anti-IgG treatment p = 2.1*10^−6^, Rx treatment p = 2.5*10^−4^. (anti-IgG  =  anti-IgG antibody, Rx  =  rituximab, Neg. controls  =  RISCfree and Luciferase-siRNA transfections, respectively; SYK knock down  =  Syk-siRNA transfections).

The cytokines CCL3/4 are easy to monitor, secreted proteins whose transcription is potently induced in response to rituxmab [31] and BCR activation. We therefore pre-incubated SUDHL4 cells with two known SYK inhibitors [32], applied the stimulatory antibodies and measured the amount of secreted CCL3/4 (Figure 5A). As expected, SYK inhibitors I and IV abrogated the CCL3/4 secretion induced by either anti-IgG antibodies or anti-CD20 antibodies.

We also silenced Syk in SUDHL4 via siRNA-mediated downregulation. Syk knockdown was confirmed by FACS analysis: Mean intensity of controls was 409 (relative light units), for Syk knockdown 262 indicating a 60% reduction of Syk surface expression after subtraction of auto fluorescence (177). This resulted in a statistically significant reduction of CCL3/4 induction of 37% and 38% for anti-BCR treatment and 25% and 28% for rituximab, respectively (Figure 5B).

Since both, direct inhibition as well as silencing of the kinase Syk abrogated antibody induced cytokine induction we concluded that BCR cascade component Syk indeed plays a role for both CD20 and BCR mediated signaling.

## Discussion

In this study, we demonstrated that anti-CD20 and anti-BCR antibodies induce similar transcriptional changes. BCR-stimulated transcription patterns were elicited in four NHL cell lines by using antibodies against the immunoglobulin part of the BCR^41^, be it IgM or IgG. Although there might be differences in the dynamics of internalization by artificially crosslinking BCRs with agonistic antibodies versus antigens that naturally crosslink the BCR [33] this seems of minor importance for our study comparing the transcriptional responses to various antibody treatments.

Treatment of cells with anti-IgM and anti-IgG antibodies induces transcription patterns related to immune response and BCR activation including components that play important roles in the BCR signaling pathway itself. A literature derived list of 61 genes for BCR activation overlaps in 16 genes for Ocily18, in 14 genes for REC1, in 30 genes for SUDHL4 and in 22 for Z138. As shown here the transcription changes induced by BCR stimulation of four cell lines have very few deregulated genes in common. This is most likely due to the different origin of the lines (Ocily18 and SUDHL4 are DLBCL, Z138 and REC1 are Mantle cell lymphoma cell lines) that reflects different developmental stages of B cells. For normal B cell development CD27 (TNFRSF7) can be used as a marker of maturity: Naïve B cells lack it, activated B and memory cells express it moderately and plasma cells express it strongly. We tried to stage the cell lines according to their expression of this and other known lineage specific CDs but as previously described in the literature [34] we found that lymphoma cell lines express marker combinations that do not fit the expression scheme for normal B cell development.

The fact that only two of four cell lines were responsive to anti-CD20 antibodies diminishes the options for comparisons, but proves that the anti-CD20 antibodies used were not inducing unspecific effects like general cytotoxicity.

Citerra el al. reported that rituximab induces different but overlapping sets of genes [35]. Interestingly, out of 16 genes described by them to be up regulated by anti-CD20 antibodies in their DHL4 cell line we found 9 (RGS2, DUSP2, IER2, NR4A1, ZFP36, FOS, ID3, ZFP36L1, CD83) to be up regulated in our data set, too.

A very interesting change in expression was observed for BIC, the locus for the miRNA155. BIC has been reported to be highly expressed and further inducible by BCR stimulation in DLBCL tumors of the activated B cell like phenotype [36]. Normally, miRNAs should not be detected by Affymetrix profiling with the HG-U133 plus 2.0 chip, but it is known that the BIC transcript has a poly A-tail [36], [37]. This explains why it was detected with our sample preparation using oligo-dT-primed cDNA synthesis.

Why some NHL cell lines are responsive to antibodies against CD20 and others are not is still a matter of debate. It has been postulated that the amount of CD20 on the cell surface influences the response to anti-CD20 antibodies as cells with high CD20 expression respond strongly, while cells with low CD20 expression do not. However some cell lines are able to respond to anti-CD20 antibody treatment only when the antibodies are hyper-crosslinked [16] indicating that responses do depend on the amount of *crosslinked* CD20. But that does not explain why cell lines bearing comparable amounts of CD20 can either be responders or not. Based on our results, we can rule out the hypothesis that cell lines non-responsive to rituximab treatment have a defect in their BCR signaling cascade, as the non-responding cell lines Ocily18 and Z138 respond to BCR-crosslinking.

Kheirallah *et al.* recently provided evidence for interaction of CD20 and BCR signaling in the proximal part of the cascade and proposed mutual inhibition of their signals [19]. Our study adds some confirmatory evidence to the latter point, since both types of treatment downregulated important components of the BCR signaling cascade. Assuming that both membrane proteins stimulate the same pathway and activate negative feedback loops by regulating the same transcriptional targets or activating inhibitory phosphatases then the pathway would be inhibited for both signals as well. Moreover Kheirallah *et al.* show that rituxmab leads to disorganization of lipid rafts and this seems to impede BCR signaling directly.

Antibodies might interact with cell surfaces not only with their specific target antigen via their CDRs but also via their generic (human) Fc-part with Fcγ receptors and one of these, FCGR2B (Fc gamma receptor 2 B), is known to interact with BCR signaling in an inhibitory manner [38]–[40] by recruiting phosphatase SHIP1 via its ITIM (immunoreceptor tyrosine-based inhibitory motif) [41], [42]. We therefore used in this study also F(ab')_2_ fragments besides whole antibodies and antibodies with Fc-portions of either human or mouse origin. Comparisons revealed varying sensitivity of cell lines with respect to this issue, maybe due to different FCGR2B expression on the cell surface or different sensitivity of the receptor [43].

A crucial part downstream in the BCR signaling cascade (that is repressed by anti-CD20 and anti-BCR antibodies in SUDHL4) is Syk. Syk inhibition blocks BCR signaling and is being investigated for the therapy of lymphoma [44]. The applied inhibitors I and IV are very specific, indicated by the fact that the reported biochemical IC50s for Syk are very low (14 nM [45] and 10 nM [46] respectively) and the IC50 of Inhibitor IV for related kinases is greater than 5 µM [45] and the reported cellular EC50s are 313 nM [45] and 110 nM [46] respectively. Direct Syk inhibition affected antibody mediated CCL3/CCL4 secretion which confirms that the signaling induced by both anti-BCR and anti-CD20 antibodies involves Syk. The same effect can be achieved by siRNA mediated silencing of Syk. It also suggests that Syk inhibitors might interfere with anti-CD20 antibody therapy.

In conclusion our study provides evidence of considerable similarities of transcription changes between anti-CD20 and anti-BCR antibodies. Combined with previous findings of interference this points to a shared pathway and provides further insight into the mode of action of anti-CD20 antibodies.

## Materials and Methods

### Cells and reagents

DLBCL cell line OciLy18 (Ontario Cancer Institute) was grown in IMDM supplemented with 20% FCS, 50 µM β-Mercaptoethanol, 25 mM Hepes und 2 mM Glutamine.

MCL cell line Z138 (Glycart) was grown in DMEM supplemented with 10% FCS and 2 mM Glutamine. MCL cell line REC-1 (DSMZ) and DLBCL cell line SUDHL-4 were grown in RPMI 1640 supplemented with 10% FCS and 2 mM Glutamine. All cell lines were cultured at 37°C in humidified 5% CO_2_ atmosphere.

For each microarray analysis 7*10^6^ cells in 7 ml suspension were treated for 4 h with 10 µg/ml whole antibody (Rituximab (Roche), LT20 (Exbio), anti-IgG antibody, anti-IgM antibody (SouthernBiotech), isotype (SouthernBiotech). To achieve equimolar concentrations we applied 6,6 µg/ml of F (ab')_2_ fragment of anti-IgM antibody (SouthernBiotech). Cells were lysed with RLTM lysis buffer (Qiagen).

### siRNA transfection

Cells were transfected by electroporation using AMAXA, Kit V (Lonza) Program O-017. Briefly 5*10^6^ cells were transfected with 1 ng SYK siRNA or control siRNAs RISC free, Luciferase (Dharmacon). Cells were seeded at 5*10^5^ cells per ml and protein depletion was assessed 40 h after transfection by FACS analysis.

### Microarray analysis

The cRNA microarray analysis was performed using genechip HG U133 Plus 2.0 (Affymetrix). Probe labeling was performed as described previously. Briefly, total mRNA was reverse transcribed and cDNA was transcribed *in-vitro* with labelled nucleotides. Labelled and fragmented cRNA was hybridized to the chip over night. Analyses were performed using GENECHIP® Operating Software (GCOS, Affymetrix). Experiments were done with three replicates.

All data are MIAME compliant and were deposited in GEO with the accession number GSE23394. http://www.ncbi.nlm.nih.gov/geo/query/acc.cgi?token=lnyxheqicsesilc&acc=GSE23394.

Data analysis comprised three steps: 1) filtering 2) consistency check via intersecting 3) characterization.

The data were filtered by applying the following criteria: at least in one of two compared samples mean >100, call >0.5, |fold change| >2.

Lists with filtered genes were compared with each another using in-house Excel ad-ins for generating unions and intersections and pictures were generated with VENNY (Oliveros J.C. (2007) VENNY. An interactive tool for comparing lists with Venn Diagrams. http://bioinfogp.cnb.csic.es/tools/venny/index.html). GO annotations were retrieved from http://david.abcc.ncifcrf.gov/tools.jsp. Further analysis was conducted using IPA – Ingenuity pathway analysis (Ingenuity Systems).

### SYK-inhibition and CCL3/4-ELISA

To quantify the amount of CCL3 released upon antibody induced BCR- or CD20-crosslinking we applied the Quantikine® Kits (Human CCL3/MIP1-alpha and Human CCL4/MIP1-beta, R&D Systems). ELISA was performed according to the manufacturer's directions. Briefly 3*10^5^ cells per well were seeded into 96well plates and pre-incubated with Syk-inhibitors I or IV (BAY61-3606) (Calbiochem) at various concentrations for 1 h. Antibodies were added and analyses were performed after additional 4 h.

### Flow cytometry/FACS

Cells were washed with cold PBS (GIBCO), and either blocked with human serum (*GIBCO*) or for intracellular target detection fixed with para-formaldehyde, permeabilized with saponine and then stained on ice for 20 min with FITC-, APC- or PE-labelled mAbs (Jackson Immunoresearch, BD Biosciences) and analysed by flow cytometry (FACS CANTO II, BD Biosciences). Analysis was performed with DIVA software (BD Biosciences).

## Supporting Information

Table S1 **Genes deregulated in SUDHL7 cells by anti-BCR and anti-CD20 treatment.** Green fill means down regulation > 2 fold, red fill up regulation > 2 fold; Symbol  =  official gene symbol.
(XLS)

Table S2 **Genes deregulated in REC1 cells: overlap between union lists for anti-BCR and anti-CD20 treatment.** Green fill means down regulation > 2 fold, red fill up regulation > 2 fold; Symbol  =  official gene symbol.
(XLS)

Table S3 **Overlapping genes in SUDHL7 and REC1 cells deregulated by anti-CD20 and anti-BCR treatment.** Green fill means down regulation > 2 fold, red fill means up regulation > 2 fold; Symbol  =  official gene symbol.
(XLS)

Table S4 **Genes deregulated by BCR stimulation in SUDHL7, REC1, Z138 and OciLy18 cells.** Green fill means down regulation > 2 fold, red fill means up regulation >2 fold; Symbol  =  official gene symbol.
(XLS)
